# The Association of Exposure to Point-of-Sale Tobacco Marketing with Quit Attempt and Quit Success: Results from a Prospective Study of Smokers in the United States

**DOI:** 10.3390/ijerph13020203

**Published:** 2016-02-06

**Authors:** Mohammad Siahpush, Raees A. Shaikh, Danielle Smith, Andrew Hyland, K. Michael Cummings, Asia Sikora Kessler, Michael D. Dodd, Les Carlson, Jane Meza, Melanie Wakefield

**Affiliations:** 1 College of Public Health, University of Nebraska Medical Center, 984365 Nebraska Medical Center, Omaha, NE 68198, USA; raees.shaikh@unmc.edu (R.A.S.); asia.sikora@unmc.edu (A.S.K.); jmeza@unmc.edu (J.M.); 2 Roswell Park Cancer Institute, Elm and Carlton Streets|Buffalo, NY 14263, USA; Danielle.Smith@RoswellPark.org (D.S.); andrew.hyland@roswellpark.org (A.H.); 3 Department of Psychiatry and Behavioral Sciences, Medical University of South Carolina, 67 President Street, MSC 861, Charleston, SC 29425, USA; cummingk@musc.edu; 4 Department of Psychology, University of Nebraska–Lincoln, 312 N 14th Street, Alexander West, Lincoln, NE 68588, USA; mdodd2@unl.edu; 5 College of Business Administration, University of Nebraska–Lincoln, 312 N 14th Street, Alexander West, Lincoln, NE 68588, USA; lcarlson3@unl.edu; 6 The Cancer Council Victoria, 615 St Kilda Road, Melbourne VIC 3094, Australia; melanie.wakefield@cancervic.org.au

**Keywords:** point-of-sale tobacco marketing, quit attempt, smoking cessation

## Abstract

The aim was to assess the association of exposure to point-of-sale (POS) tobacco marketing with quit attempt and quit success in a prospective study of smokers in the United States. Data were collected via telephone-interview on exposure to POS tobacco marketing, sociodemographic and smoking-related variables from 999 smokers in Omaha, Nebraska, in the United States. Exposure to POS tobacco marketing was measured by asking respondents three questions about noticing pack displays, advertisements, and promotions in their respective neighborhoods stores. These three variables were combined into a scale of exposure to POS tobacco marketing. About 68% of the respondents participated in a six-month follow-up phone interview and provided data on quit attempts and smoking cessation. At the six-month follow-up, 39.9% of respondents reported to have made a quit attempt, and 21.8% of those who made a quit attempt succeeded in quitting. Exposure to POS marketing at baseline was not associated with the probability of having made a quit attempt as reported at the six-month follow-up (*p* = 0.129). However, higher exposure to POS marketing was associated with a lower probability of quit success among smokers who reported to have attempted to quit smoking at six-month follow-up (*p* = 0.006). Exposure to POS tobacco marketing is associated with lower chances of successfully quitting smoking. Policies that reduce the amount of exposure to POS marketing might result in higher smoking cessation rates.

## 1. Introduction

Tobacco is one of the most marketed products in the United States [1]. The 1998 Master Settlement Agreement (MSA), which imposed significant restrictions on tobacco marketing in most outdoor places, resulted in an increased focus of the tobacco industry on marketing activities at the point of sale (POS) [2,3,4]. In 2011, the tobacco industry spent $7.5 billion on POS marketing [5] in three areas of (1) product displays; (2) advertisements; and (3) promotional and price incentives to consumers [3,6].

Despite the extensive amount of POS tobacco marketing that currently exists in the United States, very little research has addressed its impact on smoking cessation behavior. In a prospective study, Germain *et al.* examined the effect of “sensitivity” to POS tobacco displays on quitting behavior [7]. They conducted telephone surveys of 222 adult smokers in Australia at baseline and a follow-up 18 months later. Sensitivity to POS tobacco displays was measured with an index consisting of the following three variables: (1) the frequency of noticing tobacco displays; (2) unplanned purchasing behavior; and (3) deciding on brands based on POS displays. The results revealed that while sensitivity to POS marketing was not associated with making a quit attempt, lower sensitivity to POS marketing was associated with a higher probably of smoking cessation. In a qualitative study in New Zealand, Hoek *et al.* conducted semi-structured in-depth interviews with 20 participants who had attempted to quit smoking in the last six months [8]. At the time of the interview, 12 were still quit and eight had relapsed. The interviews suggested that tobacco product displays elicited emotional and physical reactions that undermined quit attempts. To our knowledge, there are no other studies that address how POS marketing influences smoking cessation. These studies only focused on a single aspect of POS tobacco marketing, namely cigarette pack displays. In the current research we employed a scale of exposure to POS tobacco marketing that included all three aspects of exposure to POS marketing (product display, advertisements, and promotional/price incentives) and examined its prospective association with quit attempt and quit success in a sample of adult smokers in a city in the United States.

## 2. Methods

### 2.1. Data and Design

This was a prospective study of a cohort of smokers in Omaha, Nebraska, who participated in a telephone interview at baseline and 6-month follow-up in the period between August 2013 and June 2014 [9]. At baseline we collected demographic, exposure to POS tobacco marketing, and smoking-related data on 999 participants using random digit dialing and placement of local advertisements in places such as the major daily newspaper and Craigslist to recruit volunteers. Our original plan was to use only random digit dialing, but this method proved to be more expensive than we had anticipated and, as a result, we sought other methods of recruitment. Those included in the study spoke English, were 18 years of age or older, had smoked more than 100 cigarettes in their life, and smoked five or more cigarettes a day at the time of the recruitment. Those who responded “never” to the following question were excluded from the study: “How often do you visit the stores in the neighborhood where you live? By stores, we mean such places as convenience stores, gas stations, grocery stores, supermarkets, drug stores, liquor stores, and tobacco stores. (never/sometimes/frequently/always)”. About 68% of respondents completed the 6-month follow-up survey and provided information on quitting behavior such as quit attempt and successful smoking cessation. The follow-up survey included questions on exposure to POS marketing as well, but we did not use these in the current research. The University of Nebraska Medical Center Institutional Review Board provided ethics approval for the study. The Institutional IRB approval number is 490-12-EP.

### 2.2. Measurement of Outcomes

Both engaging in a quit attempt and quit success were assessed at 6-month follow-up. Participants were regarded as having made a quit attempt if they responded affirmatively to the question, “Have you made any attempts to quit smoking since we last spoke with you in (month of last interview)?” [10,11,12]. Those who had made a quit attempt were asked “Are you back smoking or are you still stopped?” [13,14,15,16]. If they indicated they were still stopped, we regarded them as having successfully quit smoking. About 98% of those who reported to be still stopped had quit one week or longer prior to the interview. 

### 2.3. Measurement of Independent Variables

To measure the main independent variable POS cigarette marketing, the baseline survey asked each respondent the following questions [7,9,17,18]: “When you are in a store in your neighborhood, how often do you notice tobacco ads?”; “When you are in a store in your neighborhood, how often do you notice tobacco promotions such as special prices, multi-pack discounts, or free gift with purchase of cigarettes?”; and “When you are in a store in your neighborhood, how often do you notice cigarette pack displays?”. Possible responses to each question were: 1 = never, 2 = rarely, 3 = sometimes, 4 = often, 5 = always. The responses to the three questions were summed to create a scale of reported exposure to POS tobacco marketing with scores ranging from 3 (low marketing) to 15 (high marketing) and a Cronbach’s alpha of 0.6. This level of alpha has been considered satisfactory, especially in the early stages of research [19]. Research on the effect of cigarette marketing on smoking cessation is in its infancy. We have used this scale in previous research and have shown that it is associated with having urges to buy and impulse purchases of cigarettes [18]. 

Other independent variables, all measured at baseline, included urge to buy cigarettes, quit attempt history, motivation to quit, intention to quit, self-efficacy to quit, Heaviness of Smoking Index (HSI), sex, age, race/ethnicity, education, method of recruitment, and frequency of visits to stores in one’s neighborhood. Urge to buy cigarettes was measured with the question “When you are in a store in your neighborhood, how often do you get an urge to buy cigarettes? (1 = never; 2 = rarely; 3 = sometimes; 4 = often; 5 = always)” [17,18]. Quit attempt was measured with the question “Have you ever tried to quit smoking? (yes/no)” [20,21]. Motivation to quit was measured with the question “On a scale of one to ten, with “0” being “not at all” and “10” being “extremely”, how motivated you to give are up smoking right now?” Intention to quit was measured with the question “Are you planning to quit smoking: within the next month … within the next 6 months … sometime in the future, beyond 6 months … or not planning to quit?” [10,22,23]. Those who were planning to quit within the next six months were distinguished from those who were not. We measured self-efficacy by asking all respondents “If you decided to give up smoking completely in the next 6 months, how sure are you that you would succeed?” [22,23]. Respondents who said “somewhat sure”, “very sure” or “extremely sure” were distinguished from those who said “not at all sure” as having self-efficacy to quit. We dichotomized intention to quit and self-efficacy in order to be consistent with previous similar research using data from the United States, Canada, United Kingdom, and Australia and because the distribution of these variables was highly skewed [22,23]. HSI scores range from 0 to 6 and were calculated by summing the points for time to first cigarette after waking and number of cigarettes smoked per day. Time to first cigarette is scored as follows: <5 min = 3 points; 6–30 min = 2 points; 31–60 min = 1 point; and >60 min = 0 point. Number of cigarettes smoked per day is scored as follows: 1–10 = 0 point; 11–20 = 1 point; 21–30 = 2 points; and >31 = 3 points. Higher HSI scores indicate higher nicotine dependence [24,25]. Age was measured using the question “What is your age?”. To assess race/ethnicity, we asked the following two questions: “Are you Hispanic or Latino?” and “Which one or more of the following would you say is your race? White, Black or African American, Asian, Native Hawaiian or Other Pacific Islander, American Indian or Alaska Native, and other”. Education was measured using the question “What is the highest grade or year of school you completed?”.

### 2.4. Statistical Analysis

The quit attempt analysis included respondents who completed both baseline and follow-up interviews. The quit success analysis included only those who at follow-up indicated that they had made a quit attempt since the baseline interview. In both quit attempt and quit success analyses, we omitted observations that had a missing value for any of the analysis variables. This constituted 4.2% of the sample in the quit attempt analysis (final sample *n* = 649) and 4.7% of the sample in the quit success analysis (final sample *n* = 257). We used logistic regression models to examine the association of POS tobacco marketing with quit attempt and quit success. Independent variables whose *p*-values were greater than 0.05 in the bivariate models were not included in the multivariate models. 

In the multivariate analyses we assessed the severity of multicollinearity by examining the variance inflation factor (VIF) associated with each independent variable and found no evidence of an estimation problem arising from multicollinearity. Additionally, using Akaike Information Criterion (AIC) and Bayesian Information Criterion (BIC) for goodness-of-fit, we compared models with POS marketing as a continuous variable and models with POS marketing included as a categorical variable and found that the former provided a better fit to the data, supporting the assumption of a linear relationship between POS marketing and log odds of the outcomes.

## 3. Results

Table 1 shows the characteristics of the sample. Table 2 shows the unadjusted and adjusted effects of exposure to POS marketing and other independent variables on making a quit attempt. The unadjusted results indicated that exposure to POS marketing, urge to buy cigarettes, baseline quit attempt, motivation to quit, intention to quit, self-efficacy to quit, race/ethnicity, and method of recruitment had an association with quit attempt. In the adjusted model, exposure to POS marketing was not associated with quit attempt (*p* = 0.185). In this model, having made a quit attempt reported at baseline (*p* = 0.003), having a higher level of motivation to quit smoking (*p* < 0.001), and having an intention to quit at baseline (*p* = 0.001) were associated with a higher probability of making a quit attempt. In Supplementary Materials analyses (Table S1), we defined quit attempt as making an attempt that resulted in not smoking for longer than 24 hours. This definition did not change the conclusions of the study and there was no evidence of an association between POS marketing and quit attempt (*p* = 0.209).

**Table 1:** Sample characteristics for the quit attempt (*n* = 649) and quit success (*n* = 257) analysis.

Variables ^a^	Quit Attempt	Quit Success
	% (*n*) or Mean (Range)	% (*n*) or Mean (Range)
Quit attempt (measured at 6-month follow-up)		
Attempted	39.91 (259)	--
Did not attempt	60.09 (390)	--
Quit success (measured at 6-month follow-up)		
Succeeded	--	21.79 (56)
Did not succeed	--	78.21 (201)
Exposure to Point-of-sale marketing (POS)	8.83 (3–15)	9.37 (3–15)
Urge to buy cigarettes	2.95 (1–5)	3.14 (1–5)
Baseline quit attempt		
Attempted	83.67 (543)	91.44 (235)
Did not attempt	16.33 (106)	8.56 (22)
Motivation	4.73 (1–10)	5.74 (1–10)
Intention to quit		
Yes	31.74 (206)	46.3 (119)
No	68.26 (443)	53.7 (138)
Self-efficacy to quit		
Yes	60.55 (393)	65.76 (169)
No	39.45 (256)	34.24 (88)
HSI	3.24 (1–6)	3.22 (1–6)
Sex		
Male	40.83 (265)	39.3 (101)
Female	59.17 (384)	60.7 (156)
Age		
18–39	21.73 (141)	24.12 (62)
40–54	37.75 (245)	40.08 (103)
55+	40.52 (263)	35.8 (92)
Race/ethnicity		
Non-Hispanic White	67.95 (441)	59.92 (154)
Other	32.05 (208)	40.08 (103)
Education		
High school graduate or below	49.15 (319)	47.47 (122)
At least some college	50.85 (330)	52.53 (135)
Method of recruitment		
Random digit dialing	50.4 (327)	43.97 (144)
Other	49.6 (322)	56.03 (113)
Frequency of visits to stores		
Sometimes	12.02 (78)	10.51 (27)
Frequently	38.06 (247)	36.58 (94)
Always	49.92 (324)	52.92 (136)

**^a^** All variables were measured at baseline unless otherwise stated in the table.

**Table 2:** Logistic regression results for the effect of exposure to point-of-sale (POS) cigarette marketing and other independent variables on the odds of making a quit attempt (*n* = 649).

Independent Variables ^a^	Unadjusted Odds Ratio (95%CI)	*p*-Value	Adjusted ^b^ Odds Ratio (95%)	*p*-Value
POS cigarette marketing	1.09 (1.03–1.15)	<0.001	1.04 (0.98–1.11)	0.18
Urge to buy cigarettes	1.19 (1.06–1.34)	0.004	1.12 (0.97–1.29)	0.111
Baseline quit attempt		<0.001		0.003
Attempted	2.77 (1.7–4.54)		2.17 (1.29–3.65)	
Did not attempt	1.00		1.00	
Motivation	1.25 (1.17–1.32)	<0.001	1.14 (1.06–1.23)	<0.001
Intention to quit				0.001
Yes	3.05 (2.17–4.30)	<0.001	2.02 (1.36–3.01)	
No	1.00		1.00	
Self–efficacy to quit		<0.001		0.691
Yes	1.43 (1.03–1.98)		1.08 (0.75–1.55)	
No	1.00		1.00	
HSI	0.97 (0.81–1.15)	0.691	--	--
Sex		0.54		--
Male	0.9 (0.66–1.25)		--	
Female	1.00			
Age		0.187		--
18–39	1.00		--	
40–54	0.92 (0.61–1.4)		--	
55+	0.71 (0.67–1.08)		--	
Race/ethnicity		<0.001		0.033
Non-Hispanic White	1.00		1.00	
Other	1.84 (1.32–2.58)		1.5 (1.03–2.19)	
Education		0.395		--
High school graduate or below	1.00		--	
At least some college	1.15 (0.85–1.57)		--	
Method of recruitment		0.02		0.497
Random digit dialing	0.69 (0.5–0.94)		0.88 (0.61–1.27)	
Other	1.00		1.00	
Frequency of visits to stores		0.44		--
Sometimes	1.00		--	
Frequently	1.1		--	
Always	1.31		--	

**^a^** All independent variables were measured at baseline; **^b^** Adjusted for the effect of variables with *p* < 0.5 in the unadjusted models.

Table 3 shows the unadjusted and adjusted effects of exposure to POS marketing and other independent variables on quit success. The unadjusted results indicated that exposure to POS marketing and self-efficacy to quit had an association with quit success. In the adjusted model, only exposure to POS marketing was associated with quit success (*p* = 0.006). A one point, increase in the scale of exposure to POS marketing was associated with a 12% decrease in the odds of quit success.

In additional Supplementary Materials analyses (Tables S2–S7), we examined the association of each of the three components of the POS marketing scale with quit attempt and quit success. Quit attempt was not associated with exposure to displays (*p* = 0.275), advertisements (*p* = 0.583), and promotions (*p* = 0.202). Quit success was associated with exposure to displays (*p* = 0.013) and advertisements (*p* = 0.01), but not with exposure to promotions (*p* = 0.249).

**Table 3:** Logistic regression results for the effect of exposure to point-of-sale (POS) cigarette marketing and other independent variables on the odds of quit success (*n* = 257).

Independent Variables ^a^	Unadjusted Odds Ratio (95%CI)	*p*-Value	Adjusted ^b^ Odds Ratio (95%)	*p*-Value
POS cigarette marketing	0.88 (0.8–0.96)	0.006	0.88 (0.8–0.96)	0.006
Urge to buy cigarettes	0.95 (0.76–1.19)	0.657	--	--
Baseline quit attempt		0.911		--
Attempted	0.94 (0.33–2.68)		--	
Did not attempt	1.00		--	
Motivation	1.07 (0.95–1.19)	0.251	--	--
Intention to quit		0.353		--
Yes	1.32 (0.73–2.4)		--	
No	1.00		--	
Self–efficacy to quit		0.044		0.052
Yes	1.97 (0.66–3.90)		1.99 (0.99–3.97)	
No	1.00		1.00	
HSI	0.93 (0.67–1.3)	0.686	--	--
Sex		0.538		--
Male	1.21 (0.66–2.2)		--	
Female	1.00		--	
Age		0.164		--
18–39	1.00		--	
40–54	1.6 (0.68–3.74)		--	
55+	2.2 (0.94–5.1)		--	
Race/ethnicity		0.089		--
Non-Hispanic White	1.00		--	
Other	0.58 (0.31–1.1)		--	
Education		0.9		--
High school graduate or below	1.00		--	
At least some college	0.96 (0.53–1.74)		--	
Method of recruitment		0.103		--
Random digit dialing	1.64 (0.9–2.98)		--	
Other	1.00		--	
Frequency of visits to stores		0.2		--
Sometimes	1.00		--	
Frequently	2.2		--	
Always	1.36		--	

**^a^** All independent variables were measured at baseline. **^b^** Adjusted for the effect of variables with *p* < 0.5 in the unadjusted models.

## 4. Discussion

In this prospective study, we examined the association of exposure to POS cigarette marketing reported at baseline with quit attempt and quit success reported at a six-month follow up. We developed an exposure to POS marketing scale which included product displays, advertisements, and promotions. We found that while exposure to POS marketing was not associated with quit attempt, higher levels of exposure to POS marketing were associated with a lower probability of successfully quitting.

The results were consistent with Germain *et al.*’s study that showed sensitivity to POS cigarette display is not associated with quit attempts but is predictive of smoking cessation [7]. The results were also consistent with Hoek *et al.* qualitative study that suggested POS cigarette displays undermine smoking cessation [8]. Of note is our finding that exposure to POS marketing was the only variable among a set of smoking-related variables including HSI and sociodemographic factors that was associated with smoking cessation. Other studies have shown higher HSI to be associated with a lower probability of smoking cessation [7,10,11]. The effect of HSI in our analysis was in the expected direction but perhaps because of our small sample size in the quit success analysis, there was insufficient power to detect an effect. In relation to the effect of other independent variables on smoking cessation, our findings were in accordance with several other studies which reported no association between smoking cessation and sex, age, socioeconomic status, ethnicity, intention to quit, self-efficacy to quit, and quit attempt [7,10,11]. However, our results were not consistent with other studies that showed a higher probability of smoking cessation to be associated with older age [26], higher levels of education, [26,27,28,29] and non-Hispanic white race/ethnicity [26,27,28,29].

A strength of the study was its prospective design where the measurement of exposure took place prior to the assessment of the outcome; exposure to POS marketing was measured at baseline and quit attempt and quit success were measured at follow up. Another strength of the study was that it developed a scale of exposure to POS marketing which included its three aspects (displays, advertisements, and promotions), instead of merely focusing on exposure to POS cigarette displays as other previous studies of the relationship between marketing and smoking cessation have done [7,8]. A weakness of the study was that the sample was not a probability sample and it may not be completely representative of the smoking population. However, we note that the sociodemographic distribution of the sample was similar to the subsample of smokers in the center city of Nebraska Metropolitan Statistical Area in the Behavioral Risk Factor Surveillance System (BRFSS) [30]. For example, the mean age in our sample was 50.3 years in our sample and 53 years in BRFSS, and the percentage of respondents with a high school diploma or a lower level of education was 49.9 in our sample and 46.3 in BRFSS. Another weakness of the study is that we did not include independent variables such as poverty or financial stress that might influence both noticing cigarette promotions such as discounts and successful smoking cessation.[11,31] Finally, because we did not ask respondents from which stores in their neighborhoods they normally purchased cigarettes, we were not able to investigate whether the effect of marketing on smoking cessation varied by the type of store.

## 5. Conclusions

There is a large evidence base about the effectiveness of population-level tobacco control policies in increasing smoking cessation rates. Policies such as increasing the price of tobacco products [32], restricting smoking in public places [33,34,35], and anti-smoking mass media campaigns [36] have been shown by numerous studies to promote smoking cessation. The current study adds to this body of knowledge by suggesting that policies that reduce the amount of exposure to POS cigarette marketing might result in higher smoking cessation rates.

## Supplementary Materials

The following are available online at www.mdpi.com/www.mdpi.com/1660-4601/13/2/203/s1. **Table S1.** Logistic regression results for the effect of exposure to point-of-sale (POS) cigarette marketing and other independent variables on the odds of making a quit attempt (defined as making an attempt that resulted not smoking for longer than 24 h) (*n* = 649). **Table S2.** Logistic regression results for the effect of exposure to point-of-sale (POS) cigarette pack displays and other independent variables on the odds of making a quit attempt (*n* = 649). **Table S3.** Logistic regression results for the effect of exposure to point-of-sale (POS) cigarette advertisements and other independent variables on the odds of making a quit attempt (*n* = 649). **Table S4.** Logistic regression results for the effect of exposure to point-of-sale (POS) cigarette promotions and other independent variables on the odds of making a quit attempt (*n* = 649). **Table S5.** Logistic regression results for the effect of exposure to point-of-sale (POS) cigarette pack displays and other independent variables on the odds of quit success (*n* = 257). **Table S6.** Logistic regression results for the effect of exposure to point-of-sale (POS) cigarette advertisements and other independent variables on the odds of quit success (*n* = 257). **Table S7.** Logistic regression results for the effect of exposure to point-of-sale (POS) cigarette promotions and other independent variables on the odds of quit success (*n* = 257).
