# Supplementary material for: The Association of Exposure to Point-of-Sale Tobacco Marketing with Quit Attempt and Quit Success: Results from a Prospective Study of Smokers in the United States
Source: Int J Environ Res Public Health. 2016 Feb 6;13(2):203. doi: 10.3390/ijerph13020203 (PMC4772223; doi:10.3390/ijerph13020203)
Supplement: Supplementary file 1 [file ijerph-13-00203-s001.pdf]

Supplementary Materials: The Association of Exposure to Point-of-Sale Tobacco Marketing with Quit Attempt and Quit Success: Results from a Prospective Study of Smokers in the United States

Mohammad Siahpush, Raees A. Shaikh, Danielle Smith, Andrew Hyland, Kenneth Michael Cummings, Asia Sikora Kessler, Michael D. Dodd, Les Carlson, Jane Meza and Melanie Wakefield

Table S1. Logistic regression results for the effect of exposure to point-of-sale (POS) cigarette marketing and other independent variables on the odds of making a quit attempt (defined as making an attempt that resulted not smoking for longer than 24 h) ($n = 649$).

Independent Variables [a]	Unadjusted Odds Ratio (95%CI)	*p*-Value	Adjusted [b] Odds Ratio (95%)	*p*-Value
POS cigarette marketing	1.09 (1.03–1.15)	<0.001	1.04 (0.98–1.11)	0.209
Urge to buy cigarettes	1.19 (1.06–1.34)	0.004	1.12 (0.97-1.31)	0.107
Baseline quit attempt		<0.001		0.010
Attempted	2.77 (1.7–4.54)		2.01 (1.18–3.41)	
Did not attempt	1.00		1.00	
Motivation	1.25 (1.17–1.32)	<0.001	1.52 (1.07–1.24)	<0.001
Intention to quit				0.002
Yes	3.05 (2.17–4.30)	<0.001	1.9 (1.27–2.86)	
No	1.00		1.00	
Self-efficacy to quit		<0.001		0.445
Yes	1.43 (1.03–1.98)		1.16 (0.80–1.68)	
No	1.00		1.00	
HSI	0.97 (0.81–1.15)	0.691	--	--
Sex		0.54		--
Male	0.9 (0.66–1.25)		--	
Female	1.00			
Age		0.187		--
18–39	1.00		--	
40–54	0.92 (0.61–1.4)		--	
55+	0.71 (0.67–1.08)		--	
Race/ethnicity		<0.001		0.059
Non-Hispanic White	1.00		1.00	
Other	1.84 (1.32–2.58)		1.45 (0.98–2.14)	
Education		0.395		--
High school graduate or below	1.00		--	
At least some college	1.15 (0.85–1.57)		--	
Method of recruitment		0.02		0.527
Random digit dialing	0.69 (0.5–0.94)		0.88 (0.61–1.29)	
Other	1.00		1.00	
Frequency of visits to stores		0.44		--
Sometimes	1.00		--	
Frequently	1.1		--	
Always	1.31		--	

[a] All independent variables were measured at baseline; [b] Adjusted for the effect of variables with $p < 0.5$ in the unadjusted models.

Table S2. Logistic regression results for the effect of exposure to point-of-sale (POS) cigarette pack displays and other independent variables on the odds of making a quit attempt (n = 649).

Independent Variables [a]	Unadjusted Odds Ratio (95%CI)	p-Value	Adjusted [b] Odds Ratio (95%)	p-Value
POS cigarette pack displays	1.09 (1.03–1.15)	<0.001	1.07 (0.95–1.21)	0.275
Urge to buy cigarettes	1.19 (1.06–1.34)	0.004	1.13 (0.98–1.3)	0.08
Baseline quit attempt		<0.001		0.003
Attempted	2.77 (1.7–4.54)		2.18 (1.3–3.66)	
Did not attempt	1.00		1.00	
Motivation	1.25 (1.17–1.32)	<0.001	1.14 (1.06–1.23)	<0.001
Intention to quit				0.001
Yes	3.05 (2.17–4.30)	<0.001	2.01 (1.35–2.99)	
No	1.00		1.00	
Self-efficacy to quit		<0.001		0.651
Yes	1.43 (1.03–1.98)		1.08 (0.76–1.56)	
No	1.00		1.00	
HSI	0.97 (0.81–1.15)	0.691	--	--
Sex		0.54		--
Male	0.9 (0.66–1.25)		--	
Female	1.00			
Age		0.187		--
18–39	1.00		--	
40–54	0.92 (0.61–1.4)		--	
55+	0.71 (0.67–1.08)		--	
Race/ethnicity		<0.001		0.027
Non-Hispanic White	1.00		1.00	
Other	1.84 (1.32–2.58)		1.52 (1.05–2.22)	
Education		0.395		--
High school graduate or below	1.00		--	
At least some college	1.15 (0.85–1.57)		--	
Method of recruitment		0.02		0.381
Random digit dialing	0.69 (0.5–0.94)		0.85 (0.59–1.22)	
Other	1.00		1.00	
Frequency of visits to stores		0.44		--
Sometimes	1.00		--	
Frequently	1.1		--	
Always	1.31		--	

[a] All independent variables were measured at baseline; [b] Adjusted for the effect of variables with $p < 0.5$ in the unadjusted models.

Table S3. Logistic regression results for the effect of exposure to point-of-sale (POS) cigarette advertisements and other independent variables on the odds of making a quit attempt (n = 649).

Independent Variables [a]	Unadjusted Odds Ratio (95%CI)	p-Value	Adjusted [b] Odds Ratio (95%)	p-Value
POS cigarette advertisements	1.09 (1.03–1.15)	<0.001	1.04 (0.91–1.17)	0.583
Urge to buy cigarettes	1.19 (1.06–1.34)	0.004	1.14 (1–1.32)	0.053
Baseline quit attempt		<0.001		0.003
Attempted	2.77 (1.7–4.54)		2.19 (1.3–3.68)	
Did not attempt	1.00		1.00	
Motivation	1.25 (1.17–1.32)	<0.001	1.15(1.07–1.23)	<0.001
Intention to quit				0.001
Yes	3.05 (2.17–4.30)	<0.001	2.02 (1.36–3.01)	
No	1.00		1.00	

Table S3. *Cont.*

Independent Variables [a]	Unadjusted Odds Ratio (95%CI)	*p*-Value	Adjusted [b] Odds Ratio (95%)	*p*-Value
Self-efficacy to quit		<0.001		0.712
Yes	1.43 (1.03–1.98)		1.07 (0.75–1.54)	
No	1.00		1.00	
HSI	0.97 (0.81–1.15)	0.691	--	--
Sex		0.54		--
Male	0.9 (0.66–1.25)		--	
Female	1.00			
Age		0.187		--
18–39	1.00		--	
40–54	0.92 (0.61–1.4)		--	
55+	0.71 (0.67–1.08)		--	
Race/ethnicity		<0.001		0.036
Non-Hispanic White	1.00		1.00	
Other	1.84 (1.32–2.58)		1.5 (1.03–2.2)	
Education		0.395		--
High school graduate or below	1.00		--	
At least some college	1.15 (0.85–1.57)		--	
Method of recruitment		0.02		0.386
Random digit dialing	0.69 (0.5–0.94)		0.85 (0.59–1.22)	
Other	1.00		1.00	
Frequency of visits to stores		0.44		--
Sometimes	1.00		--	
Frequently	1.1		--	
Always	1.31		--	

[a] All independent variables were measured at baseline; [b] Adjusted for the effect of variables with $p < 0.5$ in the unadjusted models.

Table S4. Logistic regression results for the effect of exposure to point-of-sale (POS) cigarette promotions and other independent variables on the odds of making a quit attempt ($n = 649$).

Independent Variables [a]	Unadjusted Odds Ratio (95%CI)	*p*-Value	Adjusted [b] Odds Ratio (95%)	*p*-Value
POS cigarette promotions	1.09 (1.03–1.15)	<0.001	1.09 (0.95–1.25)	0.202
Urge to buy cigarettes	1.19 (1.06–1.34)	0.004	1.14 (1–1.31)	0.055
Baseline quit attempt		<0.001		0.003
Attempted	2.77 (1.7–4.54)		2.19 (1.3–3.67)	
Did not attempt	1.00		1.00	
Motivation	1.25 (1.17–1.32)	<0.001	1.15 (1.07–1.23)	<0.001
Intention to quit				0.001
Yes	3.05 (2.17–4.30)	<0.001	2 (1.34–2.96)	
No	1.00		1.00	
Self-efficacy to quit		<0.001		0.710
Yes	1.43 (1.03–1.98)		1.07 (0.75–1.54)	
No	1.00		1.00	
HSI	0.97 (0.81–1.15)	0.691	--	--
Sex		0.54		--
Male	0.9 (0.66–1.25)		--	
Female	1.00			
Age		0.187		--
18–39	1.00		--	
40–54	0.92 (0.61–1.4)		--	
55+	0.71 (0.67–1.08)		--	

Table S4. *Cont.*

**Independent Variables** [a]	**Unadjusted Odds Ratio (95%CI)**	***p*-Value**	**Adjusted** [b] **Odds Ratio (95%)**	***p*-Value**
Race/ethnicity		<0.001		0.023
Non-Hispanic White	1.00		1.00	
Other	1.84 (1.32–2.58)		1.54 (1.06–2.25)	
Education		0.395		--
High school graduate or below	1.00		--	
At least some college	1.15 (0.85–1.57)		--	
Method of recruitment		0.02		0.426
Random digit dialing	0.69 (0.5–0.94)		0.86 (0.60–1.23)	
Other	1.00		1.00	
Frequency of visits to stores		0.44		--
Sometimes	1.00		--	
Frequently	1.1		--	
Always	1.31		--	

[a] All independent variables were measured at baseline; [b] Adjusted for the effect of variables with $p < 0.5$ in the unadjusted models.

Table S5. Logistic regression results for the effect of exposure to point-of-sale (POS) cigarette pack displays and other independent variables on the odds of quit success ($n = 257$).

**Independent Variables** [a]	**Unadjusted Odds Ratio (95%CI)**	***p*-Value**	**Adjusted** [b] **Odds Ratio (95%)**	***p*-Value**
POS cigarette pack displays	0.88 (0.8–0.96)	0.006	0.77 (0.63–0.94)	0.013
Urge to buy cigarettes	0.95 (0.76–1.19)	0.657	--	--
Baseline quit attempt		0.911		--
Attempted	0.94 (0.33–2.68)		--	
Did not attempt	1.00		--	
Motivation	1.07 (0.95–1.19)	0.251	--	--
Intention to quit		0.353		--
Yes	1.32 (0.73–2.4)		--	
No	1.00		--	
Self-efficacy to quit		0.044		0.061
Yes	1.97 (0.66–3.90)		1.94 (0.97–3.86)	
No	1.00		1.00	
HSI	0.93 (0.67–1.3)	0.686	--	--
Sex		0.538		--
Male	1.21 (0.66–2.2)		--	
Female	1.00		--	
Age		0.164		--
18–39	1.00		--	
40–54	1.6 (0.68–3.74)		--	
55+	2.2 (0.94–5.1)		--	
Race/ethnicity		0.089		--
Non-Hispanic White	1.00		--	
Other	0.58 (0.31–1.1)		--	
Education		0.9		--
High school graduate or below	1.00		--	
At least some college	0.96 (0.53–1.74)		--	
Method of recruitment		0.103		--
Random digit dialing	1.64 (0.9–2.98)		--	
Other	1.00		--	
Frequency of visits to stores		0.2		--
Sometimes	1.00		--	
Frequently	2.2		--	
Always	1.36		--	

[a] All independent variables were measured at baseline; [b] Adjusted for the effect of variables with $p < 0.5$ in the unadjusted models.

Table S6. Logistic regression results for the effect of exposure to point-of-sale (POS) cigarette advertisements and other independent variables on the odds of quit success (n = 257).

Independent Variables [a]	Unadjusted Odds Ratio (95%CI)	*p*-Value	Adjusted [b] Odds Ratio (95%)	*p*-Value
POS cigarette advertisements	0.88 (0.8–0.96)	0.006	0.77 (0.63–0.94)	0.010
Urge to buy cigarettes	0.95 (0.76–1.19)	0.657	--	--
Baseline quit attempt		0.911		--
Attempted	0.94 (0.33–2.68)		--	
Did not attempt	1.00		--	
Motivation	1.07 (0.95–1.19)	0.251	--	--
Intention to quit		0.353		--
Yes	1.32 (0.73–2.4)		--	
No	1.00		--	
Self-efficacy to quit		0.044		0.042
Yes	1.97 (0.66–3.90)		2.05 (1.02–4.1)	
No	1.00		1.00	
HSI	0.93 (0.67–1.3)	0.686	--	--
Sex		0.538		--
Male	1.21 (0.66–2.2)		--	
Female	1.00		--	
Age		0.164		--
18–39	1.00		--	
40–54	1.6 (0.68–3.74)		--	
55+	2.2 (0.94–5.1)		--	
Race/ethnicity		0.089		--
Non-Hispanic White	1.00		--	
Other	0.58 (0.31–1.1)		--	
Education		0.9		--
High school graduate or below	1.00		--	
At least some college	0.96 (0.53–1.74)		--	
Method of recruitment		0.103		--
Random digit dialing	1.64 (0.9–2.98)		--	
Other	1.00		--	
Frequenchy of visits to stores		0.2		--
Sometimes	1.00		--	
Frequently	2.2		--	
Always	1.36		--	

[a] All independent variables were measured at baseline; [b] Adjusted for the effect of variables with $p < 0.5$ in the unadjusted models.

Table S7. Logistic regression results for the effect of exposure to point-of-sale (POS) cigarette promotions and other independent variables on the odds of quit success (n = 257).

Independent Variables [a]	Unadjusted Odds Ratio (95%CI)	*p*-Value	Adjusted [b] Odds Ratio (95%)	*p*-Value
POS cigarette promotions	0.88 (0.8–0.96)	0.006	0.87 (0.69–1.1)	0.249
Urge to buy cigarettes	0.95 (0.76–1.19)	0.657	--	--
Baseline quit attempt		0.911		--
Attempted	0.94 (0.33–2.68)		--	
Did not attempt	1.00		--	
Motivation	1.07 (0.95–1.19)	0.251	--	--
Intention to quit		0.353		--
Yes	1.32 (0.73–2.4)		--	
No	1.00		--	
Self-efficacy to quit		0.044		0.052
Yes	1.97 (0.66–3.90)		1.97 (0.99–3.91)	
No	1.00		1.00	

Table S7. *Cont.*

Independent Variables [a]	Unadjusted Odds Ratio (95%CI)	*p*-Value	Adjusted [b] Odds Ratio (95%)	*p*-Value
HSI	0.93 (0.67–1.3)	0.686	--	--
Sex		0.538		--
Male	1.21 (0.66–2.2)		--	
Female	1.00		--	
Age		0.164		--
18–39	1.00		--	
40–54	1.6 (0.68–3.74)		--	
55+	2.2 (0.94–5.1)		--	
Race/ethnicity		0.089		--
Non-Hispanic White	1.00		--	
Other	0.58 (0.31–1.1)		--	
Education		0.9		--
High school graduate or below	1.00		--	
At least some college	0.96 (0.53–1.74)		--	
Method of recruitment		0.103		--
Random digit dialing	1.64 (0.9–2.98)		--	
Other	1.00		--	
Frequency of visits to stores		0.2		--
Sometimes	1.00		--	
Frequently	2.2		--	
Always	1.36		--	

[a] All independent variables were measured at baseline; [b] Adjusted for the effect of variables with $p < 0.5$ in the unadjusted models.

© 2016 by the authors; licensee MDPI, Basel, Switzerland. This article is an open access article distributed under the terms and conditions of the Creative Commons by Attribution (CC-BY) license (http://creativecommons.org/licenses/by/4.0/).
